# Tackling Loneliness and Isolation in Older Adults With Virtual Reality: How do We Move Forward?

**DOI:** 10.1177/23337214231186204

**Published:** 2023-07-13

**Authors:** Daniel J. Finnegan, Sarah Campbell

**Affiliations:** 1 Cardiff University, UK; 2 Play Well for Life, Surrey, UK

**Keywords:** virtual reality, mixed reality, loneliness, intervention, technology

## Abstract

Current trends in gerontology conceptualize Virtual Reality (VR) as a tool for rehabilitation, lauding its potential for cognitive rehabilitation or as an intervention to reduce cognitive function decline. However, we must take a critical stance and identify not just the potential positive impact, but also how things may go wrong without appropriate guidelines, and the need for careful design around the interaction affordances of the technology. We conducted co-discovery and co-design workshops involving expert stakeholders and older adults (*N* = 25) over a period of 6 months, involving practical activities including user personas and focus groups to understand the complexities of loneliness and identify possible solutions with VR. Based on our findings we focus our argument on two key factors in the conceptualization of loneliness: spaces, and activities which may take place within said spaces. We present our reconceptualization of VR as a tool for group activities instead of passive consumption of content and make suggestions to the community for reducing feelings of loneliness with VR.

## Introduction

**Figure 1.**
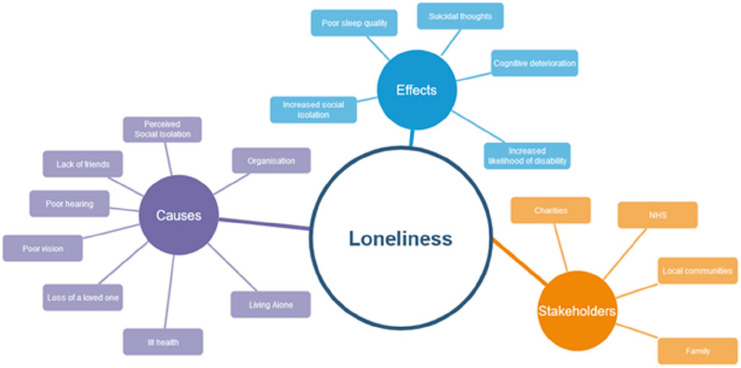
The Web of Loneliness connecting known causes arising from Loneliness, their direct effects on an individual, and second order effects on stakeholders such as family and care providers.

Loneliness is defined as “*a subjective experience of mismatch between the quality and quantity of how we perceive our social networks to be, and how we want them to be*” (Courtin & Knapp, 2017). It is an experience predicted by many interwoven factors: examples include age; gender; quality, life circumstances and breadth and depth of social networks, underpinned by (dis)possessing the necessary skills and competencies (Stuart et al., 2022; Wilson et al., 2021). It is growing as populations age with some countries taking significant steps to introduce policies and address loneliness in old age. In late life, people are more likely to have experienced the loss of a loved one and although may be resilient toward loneliness depending on their circumstance and the resources they may draw upon (Hayman et al., 2017) loneliness in later life is a rising trend. For example, in the UK the number of people experiencing loneliness over the age of 50 has increased by 49% in the last decade (Age, 2018). Over 500,000 older adults in the UK go 5 days without seeing or speaking to anyone at all, over a million report not speaking to friends, family, or neighbors for over a month and 3.9 million older adults (40%) say television is their main company (Age, 2018). Figure 1 shows our “web of loneliness”, a visualization listing several known factors involved in loneliness and their effects that it has in later life including cognitive decline.

**Figure 2.**
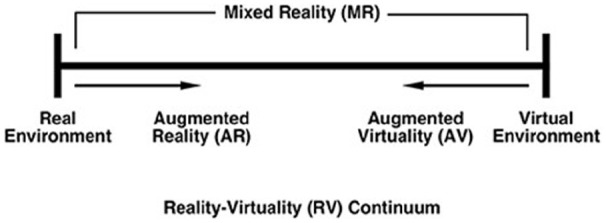
Milgram and Koshino’s Reality Virtuality Continuum which maps increasing levels of immersion mediated by a technology from the real world to a purely virtual world. Adapted from (Skarbez et al., 2021).

Technology has often been a popular choice for intervention (Baecker et al., 2014), and now using technologies as solutions to loneliness is burgeoning. Traditional interventions used external services to mitigate loneliness, often through face-to-face interactions, such as befriending services and community-based activities. With the resource-intensity of these, the recent COVID-19 pandemic stopping full or partial delivery, and the rise of common use technology in all areas of life, technology solutions are increasingly favored. Recent focus has shifted toward virtual reality technology (Figure 2). Virtual Reality (VR) refers to environments which are entirely simulated by a computer: everything you see, hear, and touch is a virtual object (Bown et al., 2017). Mixed Reality, on the other hand, combines objects which exist in the real world (Milgram et al., 1995).

Researchers and practitioners (e.g., social and care frontline workers and organizations) have tackled loneliness for decades, investigating technological interventions and studying how loneliness manifests in people of all ages, and what may be done about it with technology. At the same time, VR technology has matured to the point where compelling experiences are readily available or can be created bespoke for a given context. Given its potential for positive impact, its wide reach, and scalable deployment, we take an optimistic view on the technology and consider it to have great potential as a beneficial intervention for alleviating social loneliness in later life. We focus on the impact VR and computer simulations have on activities and spaces, and we consider the following question: “How should we use virtual reality technology to ensure it serves as an effective intervention for social loneliness in late life?” In this perspective paper, we present a synopsis of our workshops conducted with older adults in the Spring and Summer of 2022, providing details on our methodology and participant comments from interviews. We then discuss two main use cases that arose from our interview discussions with older adults and their non-prescribed use of VR: activities one may do in VR alone or with others; and places one may visit, either virtual reconstructions of real places or fantasy-based locations one may dream up. We conducted three workshops with expert stakeholders and everyday ordinary people over a 6-month period to explore how VR may be appropriated to tackle loneliness and isolation in older adults.

The first—our “Discovery” workshop—engaged stakeholders (*N* = 6, 4 female) to explore how virtual reality technology is currently used in this space and identify gaps and issues with current solutions and act as a “requirements engineering” phase of the project. Participants were domain experts from academia and third sector institutions whose mission includes reducing loneliness and feelings of isolation. The program included an icebreaker activity for participants to get to know one another, and four primary activities focused on collectively exploring the problem of loneliness in older adults: ecosystem mapping, empathy mapping, user persona construction, and user scenario ideation. All four activities are industry standard activities in service design and were used as the aim of this workshop was to develop an understanding of how digital services involving VR to tackle loneliness may be designed. Results from this workshop where that an emphasis must be placed on service delivery based around activities one could do in VR, and on one’s representation or “Avatar”—one’s visual appearance including body shape and clothing—in a virtual environment.

The second workshop took place online over three sessions—which we called “Design”—and focused on end user perspectives. Participants were older adults who had self-reported feelings of loneliness in the past 12 months in a short screening survey. These sessions were dedicated to refining the problem space and deriving a practical solution to loneliness using virtual reality. Participants (*N* = 14, 9 female) were all aged over 60 years old and were prompted 1 week before each session to prepare: a brief description of an activity they normally enjoy but feel less motivated to do when they are feeling lonely; and draw a picture of their “Avatar”—how they would like to look in VR. During sessions we chaired group discussions around what loneliness means, with participants each contributing their own personal feelings and lived experience on the topic, while focusing the discussion on activities and avatars. Specific questions we used to drive discussions on loneliness were:

- What kind of things do you do when you feel lonely?
- Who do you turn to when you feel lonely?
- Do you think modern technology has made people lonelier? If so, in what ways?
- What is the difference in your opinion between solitude and isolation, alone and lonely?

We took a grounded theory approach to analyze our notes transcribed from these sessions, using results to design several VR prototypes based on activities our participants had described. We demonstrated our prototypes in our third workshop—which we called “Inspire” (N=5, 2 female). This again involved older adults who had self-reported feelings of loneliness in the past 12 months. Participants tried our prototypes and discussed them in the context of the groups’ feelings of loneliness, with our goal being to solicit feedback on our prototypes’ appropriateness and participants’ willingness to engage with them or similar experiences in a future service delivery. In the proceeding sections, we contextualize our workshops in our discussion around VR as a tool for tackling loneliness and isolation in older adults.

## Discussion

**Figure 3.**
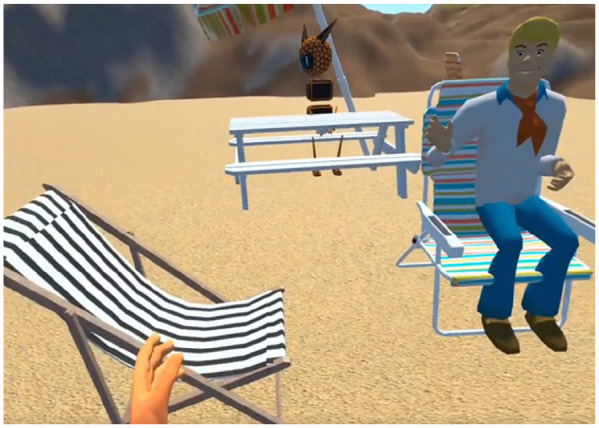
An image of a multi-user beach environment where several Avatars may socialise as requested in our interviews. Note the difference in Avatars: people are free to choose how they wish to be represented visually. We created this environment in response to our conversations with older adults and demonstrated it to them; the environment was positively received, prompting discussion, and interest in VR.

In our workshops with older adults who have experienced loneliness in a recent period, it became clear how important was the place where activities may happen. For example, one participant said they would like to “go to [sic] dance club, or small gig and would like to do virtual events like play bingo, bowling, line dancing, up to date like dancing, tea dances [sic]” as this is something they had “passed on” recently as they’ve experienced loneliness. Another said they wished they could “travel to the small village in north India that I had been to as a volunteer when I was in my early twenties,” while another said they wanted to ”fly away on a magic carpet and experience the stars up close.” These fantastical locations can be reconstructed in VR, making it possible to travel to clubs and exotic, fantasy locations. These can be tremendously empowering experiences: consider for example someone who cannot leave their house due to illness. Using VR, they may travel with friends and family to “anywhere” they like, spending as much time as they want there with others or alone (Figure 3). VR has been used extensively in the past to alleviate feelings of loneliness (Antunes et al., 2017; Hammick & Lee, 2014; Poscia et al., 2018; Veldmeijer et al., 2020), yet these have mostly focused on passive experiences (Van Houwelingen-Snippe et al., 2021), and/or reconstructions of physical social interactions for example, group meetings in an everyday space (Baker et al., 2021).

Many of the recent VR experiences produced for alleviating loneliness in older adults include reminiscence therapy, where the focus is on reliving the past rather than living the present and engaging in activities. This points to one issue in aging: “older adults” is often applied reductively as a term to capture anyone over 65 in developed countries, ignoring the vast differences in this demographic determined by health, socioeconomic status, co-morbidity, age, generation, and culture. For example, when designing technology interventions targeted at those with anxiety or depression, there are several examples of best practice including participatory design, co-creation, and data driven design (Lee et al., 2017; Randall et al., 2018). We argue that if engineers wish to engage with the field of aging to create meaningful technology solutions to prevent loneliness, it is necessary to stop thinking of anyone over 65 as falling in the category “older adult.” Instead, a dialog is necessary where both parties apply similar rigor in sample/population categorization that is seen in health and wellbeing in younger populations, beyond merely categorizing older adults by age bracket.

In our perspective, for VR to succeed the research community must work together placing emphasis along two core dimensions: activities and places, and how to appropriate VR technology for tackling loneliness across these dimensions. In the next sections we turn our discussion to each dimension in turn and provide our proposed way forward.

### Activities

Currently, most activities targeting older adults tend to be delivered face-to-face. The pandemic and the subsequent lockdowns resulted in many of these activities being canceled. The impact of this has exacerbated issues of loneliness for older people. This has been further amplified by continuous media messages about this population being most vulnerable, with problematic social media use leading to previously active and socially connected older adults suddenly finding themselves isolated and afraid to re-engage (Meshi et al., 2020). Aging residential communities have also been impacted by the increased mortality rates resulting from COVID-19 and the restrictions on how community grieving can take place (Jordan et al., 2022). The pandemic has therefore accelerated the growth in loneliness of older adults. To address this, and to be better prepared for similar future events, there is a need for service providers to embrace digital solutions and facilitate development of digital literacy where needed for those who engage with their services. One mistake seen across society during the pandemic is that offline activities and delivery can simply be translated to online delivery. However, delivering online is a different medium, with different interaction affordances and considerations, and therefore requires a redesign of service delivery.

As many of these services are delivered by charities, there is a real gap that could be addressed by interdisciplinary collaborations between technologists, user experience (UX) and human computer interaction (HCI) designers, and service providers that must be better prepared to deliver their services through technology. There is no inherent need for technology to be cutting edge either; instead, emphasis should be placed on appropriateness and the compelling nature of the use case in mind, with sufficient energy focused on ensuring backwards compatibility,^1^ integration with existing infrastructure for example, not relying heavily on 5G and future cutting-edge telecommunications, and accessible that is, easy to use by those with mobility and/or bimanual impairments.^2^ Here, the offline element of the experience becomes critical; consider how one might interact with one’s friend during a storm, when telecommunications become unreliable, and which is an overwhelmingly isolated experience in every sense of the word? We must design the offline experience with the same amount of care and attention that we give the online experience. We encourage the community to consider what activities one may perform in offline mode for example, make a move on the chess board that one’s partner will then see when upon re-establishing an internet connection, or leave a message for someone to hear for example, a note on the wall saying “Jane/Joe was here!.” These are human centered activities that shape and define our societies and culture and should be preserved in the virtual and mixed realities we build. Thus, we argue there is a need for more technology solutions facilitating human-to-human interactions, for example mixed reality and virtual reality, rather than human-to-machine/robot interactions. Perhaps robotic companions are more appropriate for those most isolated and housebound, be that because of mobility issues, personal choice, or other factors, in the first instance as a gateway to social activities with others. From this gateway, it may become appropriate to build technology solutions to facilitate human-to-human interactions to build communities that are better targeted at those less socially isolated and more mobile.

### Places

If we accept that activities and active engagement, for which VR is readily equipped to facilitate, will help in our developing new interventions for loneliness, we must now turn our attention to where such activities may take place. Place, space, and action are closely interlinked, and recent research has shown that there are clear implications for loneliness in older adults. For example, Sugiyama et al. describe the importance of local community hubs as a safe space for meeting and interacting with new people, to develop one’s social network. Two key factors they identify are convenience and comfortability, both in visiting such spaces and with respect to time spent therein (Sugiyama et al., 2022). In describing the results from their review and analysis of the literature over the past 25 years, Sugiyama et al. (2022). make several recommendations on how such spaces should look like; for example, lush greenery and visually arresting natural sights, in addition to facilitating opportunities for social interaction in the form of transition spaces for example, a bench in an urban park where one might strike up a conversation with another. They propose a research agenda focused on investigating where older adults are likely to visit for social interaction, with attention paid to proximity to the home, the function and purpose of the place for example, a community center featuring a café providing a space for social activities and food and drink, and the diversity within the population studied.

**Figure 4.**
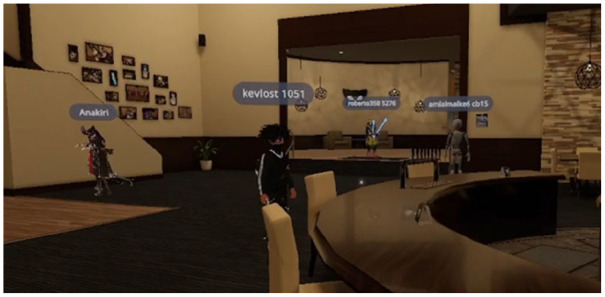
An example of a virtual club where people can meet and greet new people or old friends, or dance together and socialize.

The key point to make here is that such studies in the real world may be cumbersome. For example, large scale, longitudinal observational studies in-situ, for example, shadowing, and anthropological work, would require careful logistical planning and significant investment for travel. In addition, one has little control over day-to-day issues with weather, participant availability, and reliability of transport links and services. VR may be a significant tool in aiding such research because it immediately removes such barriers from the equation with its ability to reconstruct virtual spaces in a computer simulation. In addition, one may take a set of templated environments for example, an urban park, a café, a community center, and systematically control several variables of interest for example, the size of the space, time of day, interior and exterior décor, the types of activities on offer, who and what appear in the space; the sheer breadth of variables to manipulate make it difficult to enumerate, yet researchers have a large scope to investigate variables of interest and manipulate them as they see fit. Furthermore, such manipulation may be done with older adults as research stakeholders. In our work, we designed several virtual environments based on end user participant engagement, taking their ideas and building worlds that they would like to use (Figures 3 and 4). Participants felt valued in the research as a result, and this led to in-depth discussions that we might otherwise have missed. Using VR we can begin to address concerns raised in the literature around loneliness and the planning and design of spaces (Bagnall et al., 2023), as spaces can be designed and tested virtually with older adults experiencing loneliness, potentially opening new ways to conceptualize space and further our understanding of the links between space, social interaction affordances, and loneliness.

### Appropriating VR for Loneliness Research

Modern digital technology may rely on skills that have not been practiced by older adults for a long time or even at all, for example digital communication skills involving typing on a keyboard for example, email, browsing the internet, and in the context of VR, using a handheld controller to navigate around a virtual environment. Indeed, for most older adults, technology use remains high but there is great variation in the skills across the population (Nash, 2019) including variability in intrapersonal capacities, one’s environment, and any diagnoses made e.g., Alzheimer’s (Malinowsky et al., 2011). For many older adults, technology may remain a “black box,” something they strongly desire to engage with, but cannot begin to navigate how (Mitzner et al., 2019). This, we argue, is further evidence for an approach based on co-design and engagement for design to persuade adoption, as if applications are conveyed to older adults specifying how adopting such applications in their daily life will facilitate interactions with other people, then this may lead to higher levels of motivation amongst older adults to engage with new and emerging technology including VR.

On the other hand, we appreciate the need for caution and careful consideration as we move forward with VR. We acknowledge there remains further research to carefully balance the ability to travel to exotic, fantastical virtual worlds while considering the potential negative impact this may have on a person’s wellbeing. One need only look toward evidence from reminiscence-based interventions and their efficacy in relieving symptoms of loneliness, pointing to the group-based nature of the intervention as the significant factor, with the effect of reminiscence remaining inconclusive (Elias et al., 2020). In promoting our suggestions to leverage the possibilities brought on by VR in the context of loneliness, we remain cautious that there may perhaps be a question about whether this in fact exacerbates loneliness. As people cannot really be there, when they come back to their reality is it more painful? In the VR and HCI literature, this is a very current topic, with leading academics in their respective fields regularly debating the ethics of VR when and where it is used (Slater et al., 2020).

Ultimately, we argue for thinking, engineering, and designing for loneliness differently. Rather than target specific demographics, our opinion is that we should focus our efforts on better understanding loneliness as an “experience,” not a label: one feels lonely, not is lonely, and apply VR in our research accordingly. Recently, the Greater London Authority report on Loneliness by Mayor of London was based upon non-demographic perspectives derived from extensive analysis on the London Life Survey 2018 (Hobson & Kharicha, 2022). The report argues for operationalizing loneliness with respect to five big factors: experiencing transitions or changes in one’s established routine; acute poverty; feeling “othered” or part of an outside group; living alone or being single; being deaf and/or disabled. We see this as a step in the right direction. Rather than thinking about loneliness with respect to demographic factors—characterizing individuals with respect to age, gender—, future technological designs should emphasize facilitating interactions with others and be geared toward supporting recognized “protective shields,” and qualities which may result in feeling lonely. These include easily connecting with a support network of close family and friends, strengthening a sense of belonging in one’s community, and focusing on how technology is adopted.

In conjunction with the potential of VR, we argue that research investigating such factors may benefit from VR technology as its ability to simulate any social interaction situation in an immersive way can be leveraged to draw empathy in people. For example, one may observe firsthand a simulated scenario of moving to a large city from a rural upbringing, and study how empathy induction may lead to behavior change and reflection on our intuitive understanding of loneliness as a society. Such work would readily influence how we may act toward others who we perceive to be feeling lonely, and in turn, our conceptualization of loneliness itself. All such factors must be considered when designing VR experiences for older adults.

### Where Do We Go From Here?

VR is currently seen as a technological means to intervene when things go wrong; essentially to get things back on track. We argue this conceptualization of VR undermines its true potential. Instead, we argue for VR to be woven into the fabric of older adults’ daily lives. Instead of being something one looks to for solutions, why not have VR as any other everyday interaction paradigm. In our view, VR is no longer seen as just a targeted intervention, but a playground for innovation; an everyday tool which an individual adopts as a platform for doing activities both grand and simple. Thinking this way will break down barriers we have seen in our conversations with older adults, for example “us and them” thinking, where some have said “oh that’s for young people, it’s not for me.” Upon demonstrating the potential of the technology, with some example activities one may undertake, people’s position changed, with those we interviewed opening up to the idea and becoming excited to try the technology for themselves. We argue this will catalyze adoption, as technology is no longer seen as something to engage with for a designated purpose or in a designated setting e.g., therapy. It will encourage individuals to experiment and play with the technology, leading to new use cases not conceived at the time the technology is first presented to the older adult. For example, using a home environment originally built for virtual gardening activities as a gardening museum, inviting others in to view one’s virtual garden, share a laugh, and create memories together. The evidence in the literature supports this: successful interventions for tackling loneliness focus on shared value and common hobbies and pastimes (Williams et al., 2022).

In short, we argue for thinking about and using VR as a means of reintegration, not just rehabilitation. Our current work is exploring the design space for VR technology, focused on meaningful activities one can do alone or in groups. We are applying this conceptualization to drive several co-design workshops with older adults, affording them the opportunity to apply their own creativity and build a VR platform they want to use themselves.

## Conclusion

In this paper, we’ve discussed how virtual reality has the potential to revolutionize interventions for alleviating loneliness in older adults. We explored the question, “How should we use virtual reality technology to ensure it serves as an effective intervention for social loneliness in late life?” and in response we propose a new conceptual framework for applying VR technological interventions, one based on activities, places, and co-creation.

Of course, we acknowledge limitations and the need for caution. First, there is an unquestioned assumption we have made explicitly in this paper: is it even for the greater good? We acknowledge there remains further research to carefully balance the ability to travel to exotic, fantastical virtual worlds while considering the potential negative impact this may have on a person’s wellbeing. As we discussed earlier about leveraging the possibilities brought on by VR in the context of loneliness, we posit that there may perhaps be a question about whether this in fact exacerbates loneliness as people cannot really be there Secondly, research has suggested two main reasons people go online related to feelings of loneliness. The first describes how people go online to escape social life (displacement). When motivated this way, people’s feelings of loneliness tend to increase. The other (stimulation) describes how people go online to enrich their existing social circle or create new ones. However, things are not so simple. It remains unclear if those motivated by the perceived social benefits of online interactions (stimulation) experience more feelings of loneliness or less (Wilson et al., 2021). Perhaps this motivates bespoke VR interventions to enrich one’s social circle in a controlled way. Thirdly, VR can facilitate social interaction virtually in and around a person’s day to day routine (e.g., doing chores around the house) and even social activities (e.g., playing games). On the other hand, adoption of VR may be compromised by the perceived intention of its replacing real human contact. The latter may drive people further toward loneliness and do more harm than good. In these cases, interaction design is critical, along with understanding issues for the user relating to trust. While we believe VR technology is promising, the idea that it can fully replace interaction with a human in real life is premature. The existence of technology often presumes it must be serving a purpose, which can suit a given business case and satisfy decision makers as a cost-effective means to provide a service. However, experts in our workshop when discussing care home residents said they received the introduction of technology as an indication that nobody cared about them anymore. Others have noted great care must be taken when considering how technology is deployed in care homes (Ickert et al., 2020). Therefore, we argue for more user centered design around technological interventions aimed at reducing feelings of loneliness with older adults, letting them have a say in how technology impacts their day-to-day livelihood. We must ensure technology does not devalue the act of caring for another human being.

We have discussed the plethora of benefits arising from applying VR as a research tool, but equally raised several key issues we see in appropriating VR for loneliness—namely around questioning our assumptions on the efficacy of interventions, and the need for focused attention to known factors associated with feelings of loneliness. Given the increased capacity not just for communication but for collaboration and interaction which VR technologies provide, we call upon the community to work with engineers and technologists to establish multidisciplinary research areas to ensure solutions are robust and do not fall short or succumb yet again to the “novelty effect” and to ensure we develop truly innovative and effective interventions for tackling loneliness.

## Notes

1. Backwards compatibility is a technical term referring to new hardware and or software being interoperable with older hardware and software. For example, newer versions of popular word processing software, such as Microsoft Word, maintain a set of minimum requirements so they function on a person’s existing laptop, avoiding the need for that person to upgrade to a new laptop for as long as possible.

2. The corresponding author wishes to acknowledge insights from conversations with Tim Leech, CEO of Wavelength, a UK based charity who alleviates loneliness in rural and regionally excluded towns through the provision of analog hardware like radios and television sets.
